# Inactivation of *Morganella morganii* by high hydrostatic pressure combined with lemon essential oil

**DOI:** 10.1002/fsn3.1626

**Published:** 2020-05-12

**Authors:** Hsien‐Feng Kung, Yi‐Chen Lee, Chiu‐Chu Hwang, Ying‐Chuan Wu, Ching‐Yu Hsieh, Yung‐Hsiang Tsai

**Affiliations:** ^1^ Department of Pharmacy Tajen University Pingtung Taiwan; ^2^ Department of Seafood Science National Kaohsiung University of Science and Technology Kaohsiung Taiwan; ^3^ Department of Hospitality Management Yu Da University of Science and Technology Miaoli Taiwan

**Keywords:** high hydrostatic pressure, histamine‐forming bacteria, lemon essential oil, *Morganella morganii*, *SEM*

## Abstract

The inactivation and damage of histamine‐forming bacterium, *Morganella morganii*, in phosphate buffer and tuna meat slurry by high hydrostatic pressure (HHP) alone or in combination with 0.2% lemon essential oil (LEO) treatments were studied using viability measurement and scanning electron microscopy (*SEM*). HHP alone or in combination with LEO treatments showed first‐order destruction kinetics to *M. morganii* during pressure holding period. The D values of *M. morganii* (200 to 600 MPa) in phosphate buffer ranged from 16.4 to 0.08 min, whereas those in tuna meat slurry ranged from 51.0 to 0.10 min, respectively. *M. morganii* in tuna meat slurry had higher D values and were more resistant to HHP treatments than in phosphate buffer. In addition, the D values of HHP in combination with LEO treatment were lower than those of HHP treatment alone at <400 MPa of pressure, indicating that it is more effective to inactivate *M. morganii* under the same pressure. The results showed the *M. morganii* at HHP in combination with LEO treatment was more susceptible to pressure treatment alone. HHP with or without LEO treatments can be used to inactivate *M. morganii* by causing disruption to bacterial cell membrane and cell wall as demonstrated by *SEM* micrographs.

## Graphical abstract

*Morganella morganii* was more resistant to HHP and LEO in fish slurry than phosphate buffer. With LEO treatment, *M. morganii* was more susceptible to pressure than HHP alone. *SEM* show HHP and LEO cause cell wall and membrane damage.

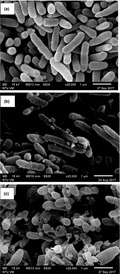

## 1 INTRODUCTION

Histamine fish poisoning, or scombroid poisoning, is an allergy‐like form of food poisoning resulting from consumption of mishandled scombroid fish that contains high contents of histamine (Lehane & Olley, 2000). Histamine is generated mainly by the decarboxylation of histidine in fish muscle through the actions of histidine decarboxylases of histamine‐forming bacteria (HFB) that are present in the seafood. HFB have been isolated from scombroid fish and other seafood products, as well as fermented foods such as wine, sausage, and cheese (Taylor, 1986). Histamine formed in fishery products is produced primarily by gram‐negative enteric bacteria. Among them, *M. morganii* has consistently been shown to form high levels of histamine (>1,000 ppm) in culture broth, and it plays the most significant role in histamine formation during the storage of fish (Kim et al., 2013).

High hydrostatic pressure (HHP) is a nonthermal technology for food pasteurization and preservation (Wang et al., 2016). In commercial setting, HHP was used at a pressure above 300 MPa to kill spoilage and pathogenic microorganisms for shelf‐life extension and safety improvement of jams, fruit juices, guacamole, meats, dairy and egg products and seafood (Considine, Kelly, Fitzgerald, Hill, & Sleator, 2008; Phuasate & Su, 2015). The usage of HHP treatment to preserve the freshness of food was also shown to not affect some of the food quality characteristics such as the color, natural flavor, and nutrients (Phuasate & Su, 2015; Singh & Ramaswamy, 2013). HHP treatment was reported to be capable of killing *Listeria monocytogenes*, *Escherichia coli*, and *Vibrio parahaemolyticus* through morphological damages to both the internal and external structures (Ramaswamy, Zaman, & Smith, 2008; Wang, Huang, Hsu, Shyu, & Yang, 2013). A treatment at a pressure of >300 MPa can cause irreversible denaturation of enzymes and proteins to affect the integrity of the cell membrane, lower protein biosynthesis, and inhibit protein repairs, and ultimately resulting in cell membrane rupture, excretion of internal substances, and bacterial death (Huang, Lung, Yang, & Wang, 2014; Wang et al., 2013).

Lemon essential oil (LEO) obtained from lemon (*Citrus lemon* L.) contains biological activity components including limonene, linalool, α‐terpineol, β‐pinene, and α‐pinene (Lin et al., 2010). LEO was reported to be capable of inhibiting food‐borne microorganisms such as *Salmonella typhimurium*, *E. coli*, and *L. monocytogenes* in media and on foods (Lin, Sheu, Hsu, & Tsai, 2010; Espina et al., 2013). Therefore, it can act as a natural preservative for improving food safety and shelf life. In addition, lemon juice and lemon fruit are extensively used as flavoring ingredients in a wide variety of foods. These ingredients are commonly added to fishes consumed raw and after cooking, especially in Asia (Lin et al., 2010). Thus, lemon aroma is well accepted for fish and the addition of LEO could be positively applied also on seafood products.

A hurdle technology is combining two or more physical or chemical preservations to inactivate spoilage and pathogenic microorganisms in foods, to lower level of chemicals (Chien et al., 2017). Recently, the inactivation effect of HHP treatment on *M. morganii* was observed using viability counting (Lee et al., 2020). Since only limited information was available on the inactivation effect and morphological damage of *M. morganii* by HHP alone and in combination with LEO treatments, the aims of this study were to find out the inactivation kinetics of HHP alone and in combination with LEO processing on *M. morganii* in 0.1 M phosphate buffer (pH 6.8) and tuna meat slurry, and to evaluate whether morphological damages occurred in HHP‐treated HFB cells.

## 2 MATERIALS AND METHODS

### 2.1 Bacterial culture and lemon essential oil preparation

Stock culture of *M. morganii* isolated from albacore tuna was kindly provided by Dr. S. H. Kim (Kim et al., 2001). It was maintained in our laboratory on Trypticase Soy Agar (Difco Becton‐Dickinson Co) at 4°C. The LEO was prepared from lemon peels according to our previous method (Lin et al., 2010). Briefly, the lemon peels (*C. lemon* L.) were diced into 1 × 1 cm pieces and stored at −20°C before extraction. The peel pieces were vacuum‐freeze dried and then ground into powder. One hundred gram of powder was placed into the supercritical CO_2_ extractor, designed by Dr. Shane‐Rong Sheu at Far East University, Tainan, Taiwan. The extraction parameters are as follows: 1.5 L capacity, temperature, 323 K; pressure, 10 MPa; flow rate of CO_2_, 3.5 kg/h; time, 90 min. Components of the extracted essential oil were analyzed according to the previous method (Lin et al., 2010) using a gas chromatograph. The major compositions of lemon essential oil were limonene (80.5%), γ‐terpinene (6.4%), β‐pipene (6.0%), and myrcene (3.5%) (data not shown).

### 2.2 Preparation of *M. morganii* in phosphate buffer and tuna meat slurry

One loopful of *M. morganii* was inoculated into Trypticase Soy Broth (TSB) tube (5 ml) and incubated at 35°C for 12 hr; then, 100 μL aliquot of the bacterial culture was added to 100 ml sterile TSB medium at 35°C for 24 hr. The cultured broth was centrifuged at 8,000 x g for 15 min at 4°C, and the bacterial pellet was washed and re‐suspended in 0.1 M phosphate buffer (pH 6.8). The bacterial suspension was then adjusted to a concentration of 10^9^ CFU/mL.

Fresh tuna flesh was purchased from a local market in Kaohsiung City, Taiwan, and transported in ice to the laboratory immediately. After washing with a 75% ethanol solution for 1 min and rinsing with sterile water, the flesh was ground to mince in a sterile food homogenizer. The fish mince was then blended with 0.1% peptone water (1:4, w/w) for 2 min in a blender (Omni International, Waterbury, CT, USA). Both the sterile phosphate buffer (0.1 M, pH 6.8, 99 ml) and the tuna meat slurry (99 ml) were inoculated with 1 ml of *M. morganii* inoculum (10^9^ CFU/mL) to get a final bacterial population of 10^7^ CFU/mL. In LEO treatments, the phosphate buffer or tuna meat slurry was added and mixed with LEO solution to get at 0.2% LEO concentration before *M. morganii* inoculation. The test samples were added to sterile vacuum bags in 10 ml portions, vacuum packaged and heat‐sealed, and then subject to HHP treatments immediately.

### 2.3 High hydrostatic pressure treatment

Test bags in triplicate were treated with a laboratory model of high pressure processing system (BaoTou KeFa, High Pressure Technology Co. Ltd) at 200 to 600 MPa for 0 to 15 min at room temperature (25°C). This high pressure processing system having a 6.2‐L chamber can be operated at up to 600 MPa at a pressure increase rate of approximately 300 MPa/min and the release times of less than 20 s at all pressures. Water was used as a pressure transmission medium in this study, and the reported pressurization times did not include the time for pressure increase or release. An untreated bag placed in ice water at ambient pressure (0.1 MPa) served as a control. Samples subject to pressure treatment were set in ice water and immediately processed for bacterial counting and *SEM* analyses.

### 2.4 Enumeration of *M. morganii* surviving cells and decimal reduction time

The HHP‐treated, HHP in combination with LEO‐treated and nontreated bacterial suspensions in phosphate buffer or fish slurry were 10‐fold serially diluted in sterile phosphate buffer (0.1 M, pH 6.8). With regard to pour plate counting, aliquots (1.0 ml) of the diluents were mixed in petri dishes with 15 ml TSA (Difco) at 45–50°C. After the agar medium was solidified in a laminar flow hood, the plates were transferred to an incubator and incubated at 30°C for 24–48 hr. Bacterial colonies numbers on the plates were counted. The detection limit of bacterial count was 1.0 log CFU/mL. Data from triplicate samples were presented as mean ± standard deviation.

The linear first‐order reaction was used as follows to determine the pressure destruction kinetics of *M. morganii* during the pressure–hold time phase with log numbers of survivors.

$$\text{Log}\;(N/N_{0})=-1/D\times t$$

where *N_0_* is the initial number of *M. morganii* in untreated samples, *N* is the surviving number of *M. morganii* after pressure treatment for time t (min)*.* The D value or decimal reduction time is the treatment time at any given pressure causing 90% reduction of the *M. morganii* population, that is,. resulting in one logarithm reduction of the microbial population. D value was obtained by the negative reciprocal slope of the log (*N/N_0_*) versus time.

The decimal logarithm of D values versus pressure was plotted to determine the pressure sensitivity of the D values, and the pressure z‐value (Zp) was determined as the negative reciprocal of the slope. The Zp is the increase of pressure needed to change the D value by 90%.

### 2.5 Scanning electron microscopy (SEM) analysis

*Morganella morganii* cells in 0.1 M phosphate buffer (pH 6.8) were harvested from pressure‐treated (500 MPa for 10 min), LEO pressure‐treated (500 MPa for 10 min), and nontreated suspensions via centrifugation at 5,000 rpm for 20 min. After two washes with phosphate buffer, the pellets were re‐suspended in 1 ml of phosphate buffer and then filtered through Millipore membranes (0.22 μm MF‐Millipore, GSWP; Millipore Corp., Billerica, MA, USA). Cells on the filters were fixed with 10 ml of 1.5% glutaraldehyde/0.1 M phosphate buffer (pH 7.3) and left overnight for drying at 4°C. After the cells on the membranes were washed three times with phosphate buffer for 10 min, they were postfixed for 90 min in 1% osmium tetroxide (OsO_4_) and then rinsed with phosphate buffer twice (10 min per rinse). The cells on the membranes were then dehydrated in a series of 10 ml ethanol solutions (35, 50, 60, 70, 85, 90, 95, 100, and 100% ethanol, 15 min each), immersed in isopentyl acetate and finally in carbon dioxide medium for critical point drying using a critical point dryer (HCP‐2, Hitachi Koki Co., Ltd., Ibaragi, Japan). The dried membranes were then mounted on scanning electron microscope stubs, sputter‐coated with a thin film of gold‐palladium and then observed by the *SEM* (S4700, Hitachi Koki Co., Ltd) operating at 15 kV voltage. *SEM* photomicrographs were taken from different regions of the same dried specimen.

### 2.6 Statistical analysis

One‐way analysis of variance (ANOVA) was carried out on the linearized survival slopes at each pressure time calculation for phosphate buffer or tuna meat slurry and was performed using the Statistical Product and Service Solutions, SPSS Version 16.0 for windows (SPSS Inc). Tukey's pairwise comparisons tests were performed within the confidence interval of 95% and value of *p* < .05 was used to indicate significant deviation.

## 3 RESULTS AND DISCUSSION

### 3.1 Inactivation kinetics of HHP treatment on *M. morganii* in phosphate buffer

**Figure 1:**
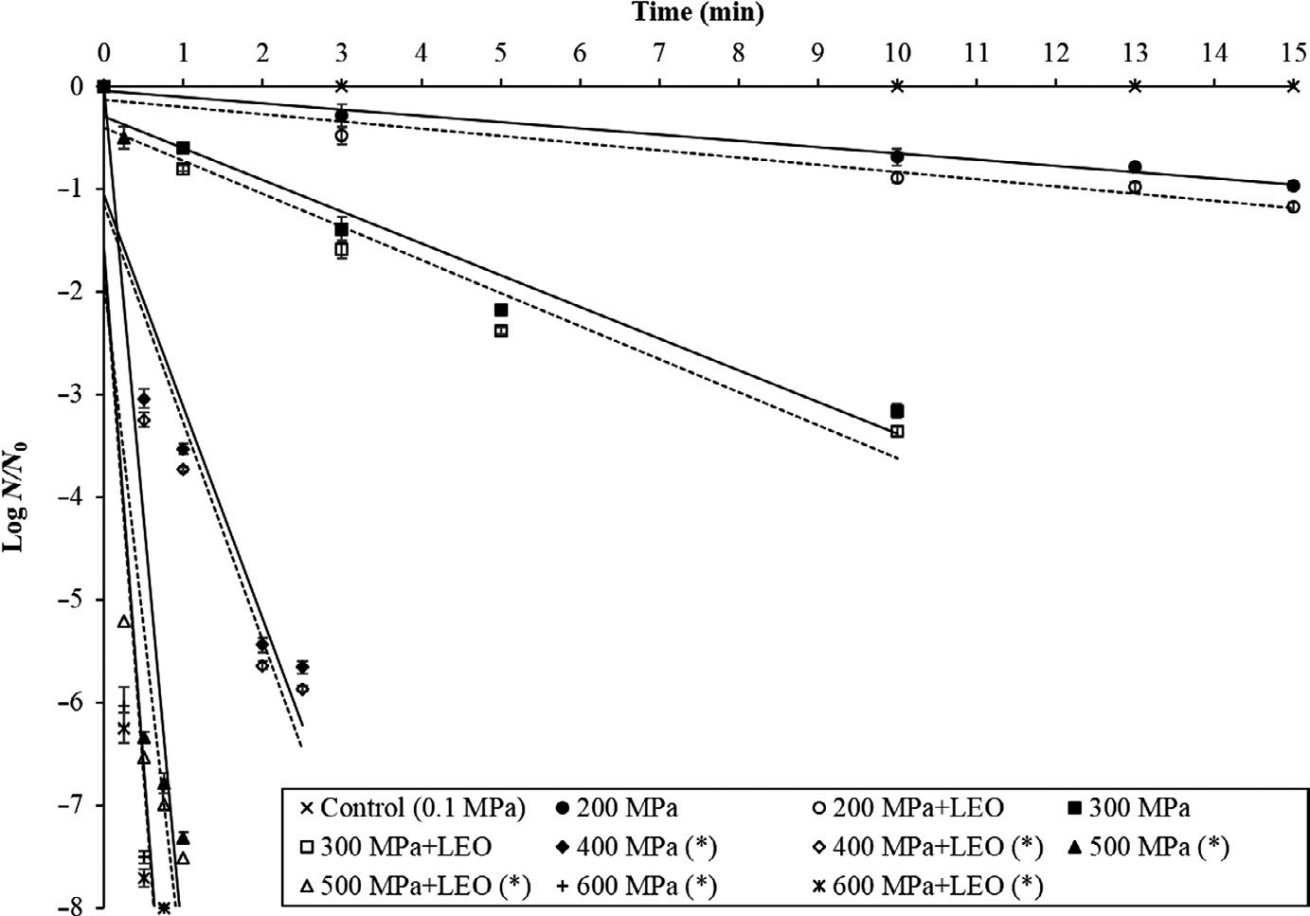
HHP survival curves of *Morganella morganii* treated with HHP treatment (200–600 MPa) for up to 15 min alone or with lemon essential oil (LEO) in phosphate buffer. (✽): No survival cell was observed in this curve at less than 15 min treatments

Figure 1 shows the survival curves of *M. morganii* in phosphate buffer following HHP treatment at 200–600 MPa for up to 15 min with or without 0.2% LEO. The treatment pressure, the LEO addition, and the holding time influenced the destruction of the bacteria. The survival curves at higher pressures were steeper than those at lower pressures indicated that the destruction rate was higher at higher pressures. The first‐order model fits the destruction kinetics of HHP treatment on *M. morganii* during the hold period, indicating that pressure destruction of *M. morganii* complied with the semi‐logarithmic model. From the survival curves, the D values could be calculated and used for comparison of microbial resistance to HHP treatments with or without LEO or the effectiveness of such treatment on microbial destruction. The D values of *M. morganii* (200 to 600 MPa) in phosphate buffer ranged from 16.4 to 0.08 min (Table 1). The computed D values of *M. morganii* in phosphate buffer showed that HHP treatments alone had higher D values (16.4 min, 3.23 min, and 0.48 min, respectively), and therefore more resistant, than HHP in combination with LEO treatments (14.2 min, 3.11 min and 0.45 min, respectively) (*p* < .05) when treated with HHP at 200, 300, and 400 MPa (Table 1). However, as the pressure level was elevated to 500 and 600 MPa, the difference in the D values diminished, with the similar D value (*p* > .05) between HHP treatments alone and HHP in combination with LEO treatments under the same pressure (Table 1). This also means that it would require shorter holding times to destroy *M. morganii* at HHP in combination with LEO treatments than HHP treatments alone at lower pressures of <400 MPa.

**Table 1:** The inactivation kinetics of HHP treatment alone or with 0.2% lemon essential oil (LEO) on *Morganella morganii* in phosphate buffer and tuna meat slurry

Pressure (MPa)	Slope	*D* value (min)^a^	*R* ^2^
In phosphate buffer			
200 MPa	−0.06	16.4 A	0.98
200 MPa + LEO	−0.07	14.2 B	0.97
300 MPa	−0.31	3.23 C	0.99
300 MPa + LEO	−0.32	3.11 D	0.99
400 MPa	−2.07	0.48 E	0.96
400 MPa + LEO	−2.12	0.45 F	0.96
500 MPa	−6.57	0.15 G	0.95
500 MPa + LEO	−6.73	0.15 G	0.95
600 MPa	−10.19	0.08 H	0.98
600 MPa + LEO	−10.18	0.08 H	0.98
In tuna meat slurry			
200 MPa	−0.02	51.0 A	0.91
200 MPa + LEO	−0.04	28.6 B	0.97
300 MPa	−0.07	13.8 C	0.99
300 MPa + LEO	−0.08	12.7 D	0.99
400 MPa	−0.60	1.67 E	0.98
400 MPa + LEO	−0.61	1.64 F	0.98
500 MPa	−1.96	0.51 G	0.91
500 MPa + LEO	−2.01	0.50 G	0.97
600 MPa	−10.21	0.10 H	0.98
600 MPa + LEO	−10.21	0.10 H	0.98

Abbreviation: *R* ^2^, regression coefficient.

^a^ D, decimal reduction time (min), values with different capital letters are significantly different (*p* < .05) within the column and the same medium.

**Figure 2:**
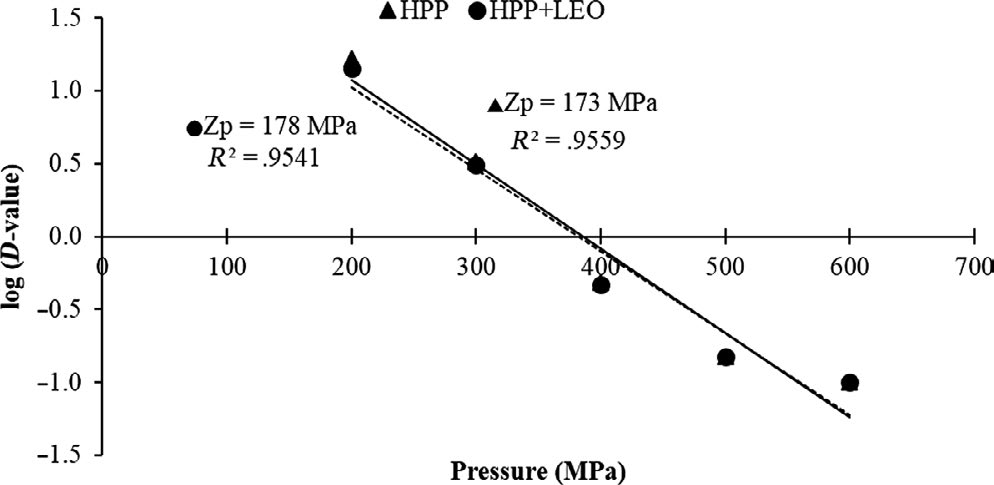
Decimal reduction time curves of HHP (200–600 MPa) alone and with lemon essential oil (LEO) for *Morganella morganii* in phosphate buffer

The HHP decimal reduction time curves as obtained by charting the decimal logarithm of D values versus pressure showed two closer regression lines in both HHP treatments alone and HHP in combination with LEO treatments (Figure 2). In phosphate buffer, the Zp value of *M. morganii* at HHP treatments alone was 173 MPa as compared with 178 MPa at HHP in combination with LEO treatments. Analysis of Zp values indicated that sensitivity of *M. morganii* to pressure changes; therefore, the destruction rate of HHP treatments alone is more sensitive to changes in pressure than HHP in combination with LEO treatments*.*

### 3.2 Inactivation kinetics of HHP treatment on *M. morganii* in tuna meat slurry

**Figure 3:**
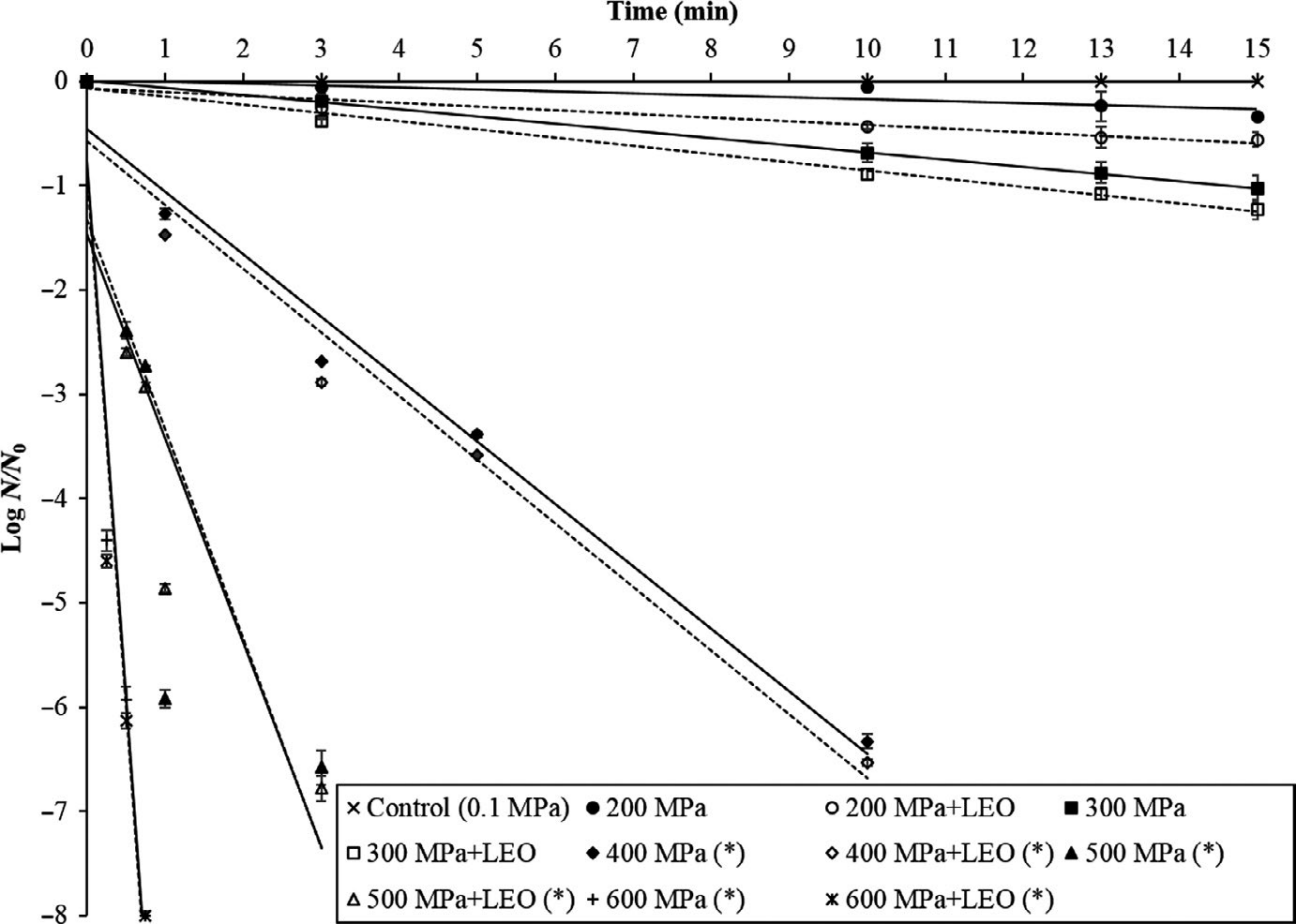
HHP survival curves of *Morganella morganii* treated with HHP treatment (200–600 MPa) for up to 15 min alone or with lemon essential oil (LEO) in tuna meat slurry. (✽): No survival cell was observed in this curve at less than 15 min treatments

The inactivation kinetics during HHP treatment (200–600 MPa for 0–15 min) with or without 0.2% LEO of *M. morganii* in tuna meat slurry is shown in Figure 3. The logarithm of the surviving *M. morganii* in the tuna slurry linearly decreased with the increase of pressure time, indicating that the HHP inactivation followed adequately first‐order kinetics. Similar to the result of phosphate buffer, the computed D values of *M. morganii* for HHP treatments alone showed a higher D value at lower treatment pressure in tuna slurry. The HHP treatments alone at 200, 300, and 400 MPa had higher D values (51.0 min, 13.8 min, and 1.67 min, respectively) than HHP in combination with LEO treatments (28.6 min, 12.7 min, and 1.64 min, respectively) (*p* < .05) (Table 1). However, as the pressure level was elevated to 500 and 600 MPa, the difference in the D values diminished, with the similar D value (*p* > .05) between HHP treatments alone and HHP in combination with LEO treatments under the same pressure (Table 1).

**Figure 4:**
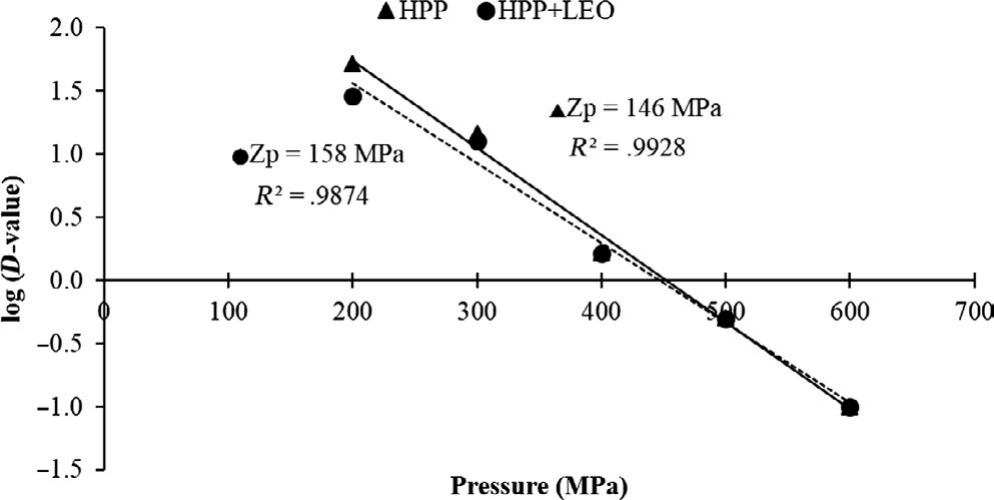
Decimal reduction time curves of HHP (200–600 MPa) alone and with lemon essential oil (LEO) for *Morganella morganii* in tuna meat slurry

The HHP decimal reduction time curves for both HHP treatments alone and HHP in combination with LEO treatments showed overlapping pressure region at 500–600 MPa (Figure 4). Thus, *M. morganii* in HHP treatments alone had a higher D value than HHP in combination with LEO treatments in tuna meat slurry, because it was more susceptible to pressure in combination with LEO, which might have accelerated the inactivation impact. In tuna meat slurry, the Zp value of *M. morganii* at HHP treatments alone was 146 MPa as compared with 158 MPa at HHP in combination with LEO treatments. Analysis of Zp values indicated that sensitivity of *M. morganii* to pressure changes; therefore, the destruction rate of HHP treatments alone is more sensitive to changes in pressure than HHP in combination with LEO treatments*.*

The combination of HHP and carvacrol was found to inactivate *L. monocytogenes* (Karatzas, Kets, Smid, & Bennik, 2001). The synergistic inactivation of *E. coli* with HHP and citral treatments was demonstrated by Chien, Sheen, Sommers, and Sheen (2017). Recently, Chien et al. (2019) reported that the combination treatment of HHP and essential oil (*Melissa officinalis*) was found to be significantly inactivated effects on *E. coli*. In this study, the D values of HHP in combination with LEO treatment were lower than those of HHP treatment alone at <400 MPa of pressure, indicating that HHP with LEO treatment is more effective to inactivate *M. morganii* under the same pressure.

In this study, the *M. morganii* in tuna meat slurry had higher D values than those in phosphate buffer for all the pressure treatment conditions (Table 1), indicating that the *M. morganii* was more resistant to pressure treatment in tuna slurry than in phosphate buffer. Many intrinsic and environmental parameters, especially the nature of the suspension medium, influence the resistance of microorganisms to pressure treatment. Simpson and Gilmour (1997) reported that bacteria existing in nutrient‐rich media had great survival ability to high pressure treatment because the media contained nutrients that are essential for repairing or substances that may provide protection against damage. Microorganisms in food systems were more resistant to HHP treatment than in buffer solution, while such resistance ability to pressure treatment increased as the water activity decreased (Cheftel & Culioli, 1997). Fish matrix was reported to have higher protective effect to spoilage bacteria than the phosphate buffer at pressures below 550 MPa (Panagou et al., 2006). Patterson (2005) also stated that some food constituents such as lipids, proteins, carbohydrates, and salt can have a protective effect for the microbial cells. Therefore, the *M. morganii* cell in tuna meat slurry are more protected against HHP treatment due to protein and lipid contents.

### 3.3 SEM micrographs of *M. morganii* after exposure to HHP treatment

**Figure 5:**
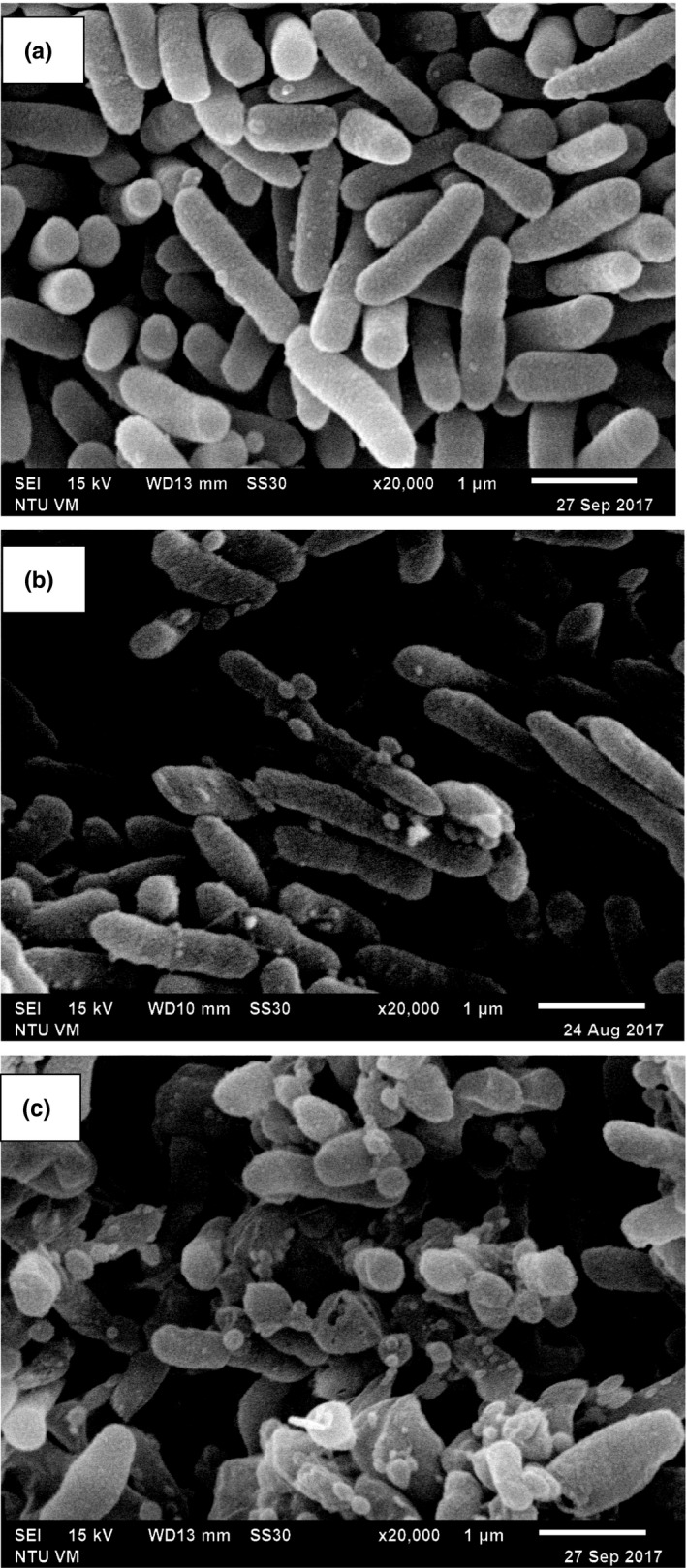
*SEM* micrographs of *Morganella morganii* cells. (a) untreated cells; (b) cells treated with 500 MPa for 10 min alone; (c) cells treated with 500 MPa for 10 min and in combination with LEO

Figure 5 is the *SEM* micrographs of *M. morganii* in phosphate buffer following HHP treatment at 500 MPa for 10 min with or without 0.2% LEO. Compared to the untreated cells (Figure 5a), damages of cellular envelopes and intracellular structures occurred with *M. morganii* after HHP treatment alone and in combination with LEO (Figure 5b and c, respectively). The HHP‐treated bacteria showed some roughness features on the cell wall, the occurrence of pimple‐like damages and swellings that resulted in some cells being compressed and other shattered (Figure 5b). Similar findings were also reported previously with the treated *Listeria* cells at 400 MPa for 10 min and *V. parahaemolyticus* at 300 MPa for 10 min (Wang et al., 2013; Pilavtepe‐Çelik et al., 2008). The observations of treated cells by HHP in combination with LEO, in Figure 5c, showed the presence of broken cell walls and perforation, and the loss of plasma membrane and cytoplasm content.

Ritz et al. (2001) employed *SEM* to show the presence of bud scars on cellular surface after pressure treatment of *L. monocytogenes*, and the loss of membrane integrity in most of the bacterial cells. Mackey, Forestiere, Isaacs, Stenning, and Brooker (1994), by using electron micrographs, showed that bacterial cells of different genera had different resistance to high pressure treatment, and pressure treatment led to changes in cellular morphology and intracellular enzyme activity. Marx et al. (2011) showed perforation damages on the cell membrane and cell wall, and scars on cells surface of *Saccharmyces cerevisiae* in apple juice after HHP treatment at 600 MPa for 7 min. Recently, Wang et al. (2013) indicated the cause of damage to bacterial membrane by HHP treatment as one of the most important underlying mechanisms of HHP inactivation of bacterial pathogens. All these studies supported the findings that HHP treatment of bacterial cells causes damages to cell membrane permeability, loss of membrane integrity, cellular swelling, and eventually cell death. In addition, it is generally known that essential oils mainly attacked the cytoplasmic membrane of bacterial cell and resulted in disturbing the structures and the increased permeability. Therefore, the cell membrane damage destroyed by essential oils may be accelerated after the HHP treatment disruption action (Chien, Sheen, Sommers, & Sheen, 2019). The results of this study suggest that the damage site of *M. morganii* by HHP treatment could be the cell membrane or cell wall, and membrane‐damaged cells may exhibit sensitivity to LEO.

## 4 CONCLUSIONS

This study, aiming of investigating the inactivation of *M. morganii* using HHP alone and with LEO treatments, showed that HHP can be applied to inactivate histamine‐forming bacterium *M. morganii* by damaging cell wall and cell membrane. The results showed that *M. morganii* in tuna meat slurry were more resistant to HHP treatment than in phosphate buffer. With LEO treatment, *M. morganii* was more susceptible to pressure treatment than HHP treatment alone*.*

## CONFLICT OF INTEREST

The authors declare no conflict of interest in the publication of this article.

## ETHICAL APPROVAL

This study does not involve any human or animal testing.

## References

[fsn31626-bib-0001] Cheftel, J. C. , & Culioli, J. (1997). Effects of high pressure on meat: A review. Meat Science, 46, 211–236. 22062123 10.1016/s0309-1740(97)00017-x

[fsn31626-bib-0002] Chien, S. Y. , Sheen, S. , Sommers, C. , & Sheen, L. Y. (2017). Modeling the inactivation of *Escherichia coli* O157: H7 and uropathogenic *E. coli* in ground beef by high pressure processing and citral. Food Control, 73, 672–680.

[fsn31626-bib-0003] Chien, S. Y. , Sheen, S. , Sommers, C. , & Sheen, L. Y. (2019). Combination effect of high‐pressure processing and essential oil (*Melissa officinalis* extracts) or their constituents for the inactivation of *Escherichia coli* in ground beef. Food and Bioprocess Technology, 12, 359–370.

[fsn31626-bib-0004] Considine, K. M. , Kelly, A. L. , Fitzgerald, G. F. , Hill, C. , & Sleator, R. D. (2008). High‐pressure processing ‐effects on microbial food safety and food quality. FEMS Microbiology Letters, 281, 1–9. 18279335 10.1111/j.1574-6968.2008.01084.x

[fsn31626-bib-0005] Espina, L. , García‐Gonzalo, D. , Laglaoui, A. , Mackey, B. M. , & Pagán, R. (2013). Synergistic combinations of high hydrostatic pressure and essential oils or their constituents and their use in preservation of fruit juices. International Journal of Food Microbiology, 161(1), 23–30. 23246609 10.1016/j.ijfoodmicro.2012.11.015

[fsn31626-bib-0006] Huang, H. W. , Lung, H. M. , Yang, B. B. , & Wang, C. Y. (2014). Responses of microorganisms to high hydrostatic pressure processing. Food Control, 40, 250–259.

[fsn31626-bib-0007] Karatzas, A. K. , Kets, E. P. W. , Smid, E. J. , & Bennik, M. H. J. (2001). The combined action of carvacrol and high hydrostatic pressure on *Listeria monocytogenes* Scott A. Journal of Applied Microbiology, 90(3), 463–469. 11298243 10.1046/j.1365-2672.2001.01266.x

[fsn31626-bib-0008] Kim, D. H. , Kim, K. , & Ahn, D. H. (2013). Inhibitory effects of high‐hydrostatic‐pressure treatments on histamine production in mackerel (*Scomber japonicus*) muscle inoculated with *Morganella morganii* and *Photobacterium phosphoreum* . Food Control, 34, 307–311.

[fsn31626-bib-0009] Kim, S. H. , Field, K. G. , Morrissey, M. T. , Price, R. J. , Wei, C. I. , & An, H. (2001). Source and identification of histamine‐producing bacteria from fresh and temperature‐abused albacore. Journal of Food Protection, 64, 1035–1044. 11456189 10.4315/0362-028x-64.7.1035

[fsn31626-bib-0010] Lee, Y. C. , Hsieh, C. Y. , Chen, M. L. , Wang, C. Y. , Lin, C. S. , & Tsai, Y. H. (2020). High‐pressure inactivation of histamine‐forming bacteria *Morganella morganii* and *Photobacterium phosphoreum* . Journal of Food Protection, 83, 621–627. 32221566 10.4315/0362-028X.JFP-19-267

[fsn31626-bib-0011] Lehane, L. , & Olley, J. (2000). Histamine fish poisoning revisited. International Journal of Food Microbiology, 58, 1–37. 10898459 10.1016/s0168-1605(00)00296-8

[fsn31626-bib-0012] Lin, C. M. , Sheu, S. R. , Hsu, S. C. , & Tsai, Y. H. (2010). Determination of bactericidal efficacy of essential oil extracted from orange peel on the food contact surfaces. Food Control, 21, 1710–1715.

[fsn31626-bib-0013] Mackey, B. M. , Forestiere, K. , Isaacs, N. S. , Stenning, R. , & Brooker, B. (1994). The effect of high hydrostatic pressure on *Salmonella thompson* and *Listeria monocytogenes* examined by electron microscopy. Letters in Applied Microbiology, 19, 429–432.

[fsn31626-bib-0014] Marx, G. , Moody, A. , & Bermúdez‐Aguirre, D. (2011). A comparative study on the structure of *Saccharomyces cerevisiae* under nonthermal technologies: High hydrostatic pressure, pulsed electric fields and thermo‐sonication. International Journal of Food Microbiology, 151, 327–337. 22015244 10.1016/j.ijfoodmicro.2011.09.027

[fsn31626-bib-0015] Panagou, E. Z. , Tassou, C. C. , Manitsa, C. , & Mallidis, G. (2006). Modelling the effect of high pressure on the inactivation kinetics of a pressure‐resistant strain of *Pediococcus damnosus* in phosphate buffer and gilt‐head seabream (*Sparus aurata*). Journal of Applied Microbiology, 102, 1499–1507. 10.1111/j.1365-2672.2006.03201.x 17578414

[fsn31626-bib-0016] Patterson, M. F. (2005). Microbiology of pressure‐treated foods. Journal of Applied Microbiology, 98, 1400–1409. 15916652 10.1111/j.1365-2672.2005.02564.x

[fsn31626-bib-0017] Phuasate, S. , & Su, Y. C. (2015). Efficacy of low‐temperature high hydrostatic pressure processing in inactivating *Vibrio parahaemolyticus* in culture suspension and oyster homogenate. International Journal of Food Microbiology, 196, 11–15. 25498471 10.1016/j.ijfoodmicro.2014.11.018

[fsn31626-bib-0018] Pilavtepe‐Çelik, M. , Balaban, M. O. , Alpas, H. , & Yousef, A. E. (2008). Image analysis based quantification of bacterial volume change with high hydrostatic pressure. Journal of Food Science, 73, 423–429. 10.1111/j.1750-3841.2008.00947.x 19021813

[fsn31626-bib-0019] Ramaswamy, H. , Zaman, S. U. , & Smith, J. P. (2008). High pressure destruction kinetics of *Escherichia coli* (O157:H7) and *Listeria monocytogenes* (Scott A) in a fish slurry. Journal of Food Engineering, 87, 99–106.

[fsn31626-bib-0020] Ritz, M. , Tholozan, J. L. , Federighi, M. , & Pilet, M. F. (2001). Morphological and physiological characterization of *Listeria monocytogenes* subjected to high hydrostatic pressure. Applied Environmental Microbiology, 67, 2240–2247. 11319107 10.1128/AEM.67.5.2240-2247.2001 PMC92862

[fsn31626-bib-0021] Simpson, R. K. , & Gilmour, A. (1997). The effect of high hydrostatic pressure on *Listeria monocytogenes* in phosphate‐buffered saline and model food systems. Journal of Applied Microbiology, 83, 181–188. 9281822 10.1046/j.1365-2672.1997.00215.x

[fsn31626-bib-0022] Singh, A. , & Ramaswamy, H. (2013). Effect of high pressure processing on color and textural properties of eggs. Journal of Food Research, 2, 11–24.

[fsn31626-bib-0023] Taylor, S. L. (1986). Histamine food poisoning: Toxicology and clinical aspects. Critical Reviews in Toxicology, 17, 91–128. 3530640 10.3109/10408448609023767

[fsn31626-bib-0024] Wang, C. Y. , Huang, H. W. , Hsu, C. P. , & .Yang, B. B., (2016). Recent advances in food processing using high hydrostatic pressure technology. Critical Reviews in Food Science and Nutrition, 56, 527–540. 25629307 10.1080/10408398.2012.745479

[fsn31626-bib-0025] Wang, C. Y. , Huang, H. W. , Hsu, C. P. , Shyu, Y. T. , & Yang, B. B. (2013). Inactivation and morphological damage of *Vibrio parahaemolyticus* treated with high hydrostatic pressure. Food Control, 32, 348–353.

